# Use of digital technologies to deliver supportive care to adolescents and young adults following treatment for cancer

**DOI:** 10.1097/SPC.0000000000000821

**Published:** 2026-10-02

**Authors:** Samantha C. Sodergren, Nicole Collaço

**Affiliations:** a School of Health Sciences, University of Southampton, Southampton, UK; b University College London Hospitals NHS Foundation Trust, London, UK

**Keywords:** adolescents and young adults, cancer survivorship, digital technologies, implementation science, supportive care

## Abstract

**Purpose of review:**

This review synthesises recent evidence on digital interventions designed to support adolescents and young adults (AYAs) following cancer treatment. It evaluates their effectiveness in addressing complex psychosocial and healthcare needs and their potential to enhance access to personalised, flexible supportive care. This is timely given improving survival rates, persistent gaps in age-appropriate follow-up, and growing interest in digital and AI-enabled care.

**Recent findings:**

Digital approaches include web-based programmes, mobile health applications, telehealth models, and emerging AI-driven tools. These interventions show promise for improving psychological outcomes, self-management, and access to support. However, the evidence base is limited, with most studies focused on feasibility and acceptability rather than efficacy. Findings are constrained by small, often unrepresentative samples, short follow-up periods, and limited evaluation of long-term outcomes and engagement.

**Summary:**

Digital technologies offer significant potential to improve AYA supportive care, but robust evidence is lacking. Most interventions adopt a ‘human-in-the-loop’ model, emphasising the continued role of healthcare professionals. High-quality trials are needed to establish clinical benefit and feasibility. Future research should prioritise sustainability, user engagement, accessibility, and integration into existing care pathways without increasing the burden on healthcare systems.

## INTRODUCTION

### Background

Adolescents and young adults (AYAs) aged 15–39 years at the time of cancer diagnosis [1], face unique long-term physical, psychosocial, and practical challenges following treatment [2–4], requiring survivorship care that is developmentally appropriate, accessible, and responsive to their evolving needs. Despite an ever- increasing number of AYAs surviving cancer [5,6] and widespread recognition that survivorship care must be tailored to the distinct, age-specific needs of AYAs, as reflected in clinical guidelines [7,8], evidence indicates sub-optimal care delivery [9–14]. Furthermore, disparities in post-treatment support are accentuated in low- and middle-income countries [15].

Digital health interventions, including mobile applications, telehealth, wearable technologies, and emerging artificial intelligence (AI)-enabled [16] and immersive virtual reality tools [17^▪▪^,^18^], offer promising approaches to delivering flexible, equitable, accessible, and personalised supportive care for this population and are recognised in recent UK health and cancer plans [19,20]. Digital innovations are increasingly being integrated into oncology care, for example, to aid diagnosis and patient decision-making [21] and offer on-demand support to reduce distress, improve self-efficacy, and improve quality of life (QoL) [22]. However, evidence regarding their role in post-treatment survivorship support for AYAs remains fragmented.

KEY POINTS

- Digital technologies (web-based programmes, mobile applications, telehealth, and emerging AI tools) have the potential to provide flexible and personalised supportive care for AYAs following cancer treatment.
- Early evidence suggests benefits in improving psychological outcomes, self-management, and access to supportive care; however, this evidence remains largely based on feasibility and acceptability studies.
- The evidence is limited by small and often unrepresentative samples, short follow-up, and limited evidence on long-term engagement and effectiveness.
- Digital interventions should be co-designed with AYAs and complement, rather than replace, support from peers and healthcare professionals.
- Robust, inclusive trials are needed to establish the effectiveness, sustainability, safety, equity, and integration into routine survivorship care.

### Scope of the review

This review summarises recent developments in digital interventions designed to support AYAs after cancer treatment, examines current evidence relating to their effectiveness, and identifies ongoing challenges and future opportunities in this rapidly evolving field.

## ONLINE INTERVENTIONS

Online interventions represent one form of digital supportive care for AYAs. Tutelman *et al*. (2024) evaluated an 8-week, co-created, online, group-based, cognitive-existential psychotherapy programme for AYAs in Canada (15–29 years) [23], covering topics such as emotional well-being, body image, and sexual health. The programme’s feasibility was tested with 33 AYAs (76% female, 70% white). AYAs indicated that opportunities to meet other AYAs and share experiences and feelings were the most valuable aspects of the intervention. The session focusing on planning for the future was rated as very useful by >70% of AYAs. While most (number not reported) AYAs reported that they would have preferred an in-person option, all indicated that the virtual delivery mode was comfortable and convenient. Overall improvements in QoL (with emotional and functional domains reaching significance) and in psychosocial outcomes, including fear of cancer recurrence, psychological distress, and body image concerns, as well as increased post-traumatic growth, were reported following the intervention. Given the small sample size and attrition, limited AYA representation, and exploratory design, the findings are not conclusive.

Zhang *et al*. (2023) piloted an 8-week coach-assisted online cognitive behavioural therapy programme, Mind Your Total Health, for the management of depression in AYAs following treatment for cancer [24]. The 10 AYAs receiving the intervention showed a significant pre- to post-intervention reduction in depression and, compared with the active control group, had significantly lower post-intervention depression. As with Tutelman *et al*.’s (2024) study [23], the evidence presented is limited by the small sample size, and thus more extensive testing is needed, as are study designs that support efficacy evaluation, help identify which AYAs are most likely to benefit, and identify the specific components that contribute to improved outcomes.

Building upon an initial pilot, proof-of-concept study of a digital health intervention, EMPOWER (Enhancing Management of Psychological Outcomes With Emotion Regulation), to reduce distress and promote well-being amongst AYAs with cancer [25], Salsman *et al*. (2024) designed [26^▪▪^] a randomised trial to determine the key components of the intervention facilitating improved psychological well-being. EMPOWER offers behavioural and cognitive skills training across five sessions with a focus on: (1) positive events, capitalising, and gratitude; (2) mindfulness; (3) positive reappraisal; (4) personal strengths and goal setting; and (5) acts of kindness. This work also contributes to precision supportive care by offering a mechanism to identify AYAs most at risk of psychological distress. Trial outcomes at baseline, during the intervention, post-intervention, and 2- and 4-month follow-up include positive affect, emotional health, adherence, retention, physical functioning, fatigue, satisfaction with social roles and responsibilities, global physical health, and health behaviours. Mediating and moderating variables explored include self-efficacy (general and coping), social support, weekly stressors, and positive events. The results of this trial are pending; the design offers a more rigorous evaluation of digital supportive care.

ASCENT (AYA Survivors Coping and Emotional Needs Toolkit) [27] is another example of an online intervention designed to promote self-management of depression amongst AYAs following treatment and is delivered over a 6-week period. Nested within this tool are daily mood tracking, a psychoeducational module about cancer and depression, and four components based on evidence-based interventions for depression. The toolkit is currently being piloted with 16 AYAs who have finished cancer treatment in the past 5 years to evaluate its feasibility and acceptability, as well as to identify which components meaningfully contribute to improved depressive symptoms. This work is also compromised by its modest sample size.

While the online interventions above address the psychological support needs of AYAs, the PREVENT web-based clinical support platform [28] focuses on behaviour change within a subset of ‘at risk’ AYAs with cancer and supports health care teams to deliver health behaviour counselling with respect to physical activity, food intake, and cardiovascular health. A randomised controlled pilot study involving 55 AYAs (12–39 years), who were overweight or obese and at least 6 months after active cancer treatment, demonstrated improvements in self-efficacy, physical activity, and healthy food intake [29^▪▪^].

## MOBILE HEALTH TECHNOLOGIES: SOFTWARE APPLICATIONS (‘APPS’) DESIGNED FOR SMARTPHONES OR TABLETS

A first-of-its-kind, co-designed app (Kræftværket) [30], providing an online social platform, is currently being rolled out for AYAs (15–39 years) in Denmark to foster a sense of connectedness and reduce isolation. A pilot study with 20 AYAs indicated improvements in QoL following 6 weeks of use [31]. A subsequent study capturing questionnaire and follow-up interview feedback from 85 AYAs (15–29 years) indicated that the app was not necessarily used daily and was likely to be more relevant for AYAs on treatment [32^▪▪^]. However, 26% of participants in follow-up care indicated that they would want to be part of the forum after the follow-up period, suggesting a sustained need for the peer support offered by the app. Differential engagement by gender, with greater use among women than men, also highlights the need to better understand barriers to uptake across subgroups.

These findings represent an important step toward implementation, demonstrating both feasibility and acceptability of digitally delivered peer support for AYAs with cancer. However, the evidence remains preliminary, with small sample sizes, short follow-up periods, and limited understanding of long-term engagement, clinical effectiveness, and equity of access.

Jiang *et al*. (2024) evaluated a mobile app incorporating AI features to address cancer-related symptoms and improve psychological health in women aged 18–45 years treated for breast cancer in China [33^▪^]. The women were randomly allocated to a control (*n* = 62) or intervention (*n* = 62) arm. The intervention included access to the app (AI-TA) comprising several modules to support various aspects of survivorship, including psychoeducation components in different formats. The app’s design allowed for the generation of recommendations and content delivery tailored to individuals’ needs, data monitoring and evaluation by healthcare professionals (HCPs), and peer-to-peer engagement to share experiences and advice. Thus, the app offers supportive, relevant, interactive, and dynamic capabilities. The intervention was delivered over a 3-month period and was also supplemented with fortnightly web-based follow-ups via private messaging or calls, providing the women with a secure and supportive platform to share aspects of their daily lives and to co-create a health plan moving forward, driven by their goals, resources, and needs. Participants were also invited to contact HCPs between calls. In the control group, women were given access to the mobile app and its modules but did not benefit from the app’s intelligent features and were not invited to participate in forums. Significant improvements in psychological symptoms and enhanced self-efficacy were observed for the intervention group, while improvements in social support and QoL were not evident until the 3-month follow-up. The authors noted that the small sample size and heterogeneity of the treatment profiles of the young women could have compromised the study’s power. Furthermore, the study was conducted within a relatively small timeframe; thus, long-term effects and sustainability of the intervention remain unexplored. Generalisability of findings to other cancer types and cultures is also limited.

## TELEHEALTH INTERVENTIONS

Stepped-care remote consultations with HCPs added to a personalised digital survivorship program, INSPIRE (INteractive Survivorship Program to Improve health care REsources), are being tested as part of a risk-stratified, randomised controlled trial to improve healthcare adherence and reduce cancer-related distress amongst AYAs (15–39 years and 1–5 years post-diagnosis) [34^▪^]. The intervention introduces a telehealth component (stepped care) to digital methods (mobile app, study website, social media) for AYAs identified at the 6-week screening as having elevated distress or depression, poor self-management literacy, and low digital resource engagement. The telehealth component involves phone calls to support goal-setting and promote problem-solving. Its multimodal design may address diverse AYA supportive care needs post-treatment, but effectiveness, accessibility, cost-effectiveness, long-term engagement, and scalability remain unproven.

## CONVERSATIONAL AGENTS

AI-driven chatbots and virtual assistants can enhance supportive care by providing real-time monitoring of symptoms, education, and psychosocial support, although there is limited evidence to support long-term engagement with such agents and psychological benefits for people with cancer, irrespective of age [35]. Furthermore, AI can support the development of personalised and adaptive self-care interventions by learning from and responding to the individual user’s symptoms and functionality, as well as preferences, over time [36]. Such technology is particularly appealing for use with individuals facing geographic or socioeconomic barriers to traditional care.

In paediatric and AYA oncology, Puthenpura *et al*. [17^▪▪^] are piloting an AI-patient navigator that provides personalised support and real-time information, and translates medical jargon. The navigator’s age-appropriate communication style is modelled on the writing and personality styles adopted by users of AYA social media accounts. Again, evidence to support this technology has not yet been established.

## AYA FEEDBACK ON DIGITAL INTERVENTIONS

Mostafa *et al*. (2025) systematically reviewed qualitative studies of AYAs’ (15–39 years) experiences with digital cancer supportive care interventions [37^▪▪^]. Desirable attributes identified included age-appropriate content, flexible options, intuitive technology, and safe, inclusive spaces fostering peer connection. Challenges included feeling overwhelmed, disengagement with inaccessible or irrelevant content, and difficulty finding private spaces for online participation.

Digital forums helped AYAs feel connected, but some preferred opportunities for in-person meet-ups and more direct, two-way communication with HCPs to complement digital support.

## SYNTHESIS OF EVIDENCE

While there is no shortage of proposed digital solutions for delivering AYA supportive care, most interventions focus on the early phase of the cancer trajectory, during active treatment [38,39]. In contrast, programs specifically designed for AYAs post-treatment remain limited and are largely still at the pilot or feasibility stage, meaning that evidence regarding their efficacy is either not yet available or insufficiently robust to support firm conclusions about their effectiveness, thereby impeding their broader implementation into routine clinical practice. Follow-up periods for AYAs participating in these interventions are typically short, leaving the sustainability of any observed benefits unclear. In addition, many studies are underpowered, with small sample sizes and suboptimal retention rates limiting the strength and reliability of their findings.

The studies presented in this review focus on the interventions’ feasibility and acceptability, with promising results, and underline the importance of involving AYAs in the development process [30]. In addition, although delivered through digital and remote technologies, these interventions continue to rely on meaningful human input and interaction [23,34^▪^] rather than functioning as entirely automated solutions. Furthermore, HCPs serve as important advocates and key partners in their delivery [28]. Although these technologies are flexible and asynchronous and meet AYAs where they are [14], the importance of in-person support and peer connections is highlighted [23,33^▪^,^34^^▪^].

A key motivation for the development and subsequent adoption of these technologies is to help address health inequalities related to cultural, socioeconomic, linguistic, and geographical barriers [17^▪▪^]. However, the evaluations presented can be criticised for limited testing among marginalised and underrepresented populations; thus, their potential for widespread adoption remains uncertain.

Despite some evidence and commentary on ethical and safety concerns for digital technologies in AYA cancer care, these issues are typically mentioned as broad concerns (e.g., ensuring age-appropriate content, risk management) rather than being rigorously evaluated [40].

## CONCLUSION

Digital technologies hold promise for delivering personalised, flexible, equitable, and high-quality care to AYAs with cancer, particularly during the post-treatment phase, when support needs may intensify as the safety net of regular healthcare contact is withdrawn, and many feel disoriented or overwhelmed. Realising this potential, however, requires robust evidence of efficacy from well-designed prospective and cross-cultural trials that adopt inclusive recruitment strategies, alongside efforts to address implementation barriers related to governance, safety, and ethics. Ultimately, such technologies are likely to be most effective when positioned as an adjunct to, rather than a replacement for, in-person support.
